# Numb-dependent integration of pre-TCR and p53 function in T-cell precursor development

**DOI:** 10.1038/cddis.2014.438

**Published:** 2014-10-16

**Authors:** N M Martin-Blanco, S Checquolo, F Del Gaudio, R Palermo, G Franciosa, L Di Marcotullio, A Gulino, M Canelles, I Screpanti

**Affiliations:** 1 Department of Molecular Medicine, Sapienza University, 00161 Rome, Italy; 2 Department of Medico-Surgical Sciences and Biotechnology, Sapienza University, Latina 04100, Italy; 3 Center for Life Nano Science@Sapienza, Istituto Italiano di Tecnologia, Rome 00161, Italy; 4 Neuromed Institute, Pozzilli 86007, Italy; 5 Institute of Parasitology and Biomedicine ‘Lopez Neyra', Spanish National Research Council, Granada 18100, Spain; 6 Institut Pasteur-Foundation Cenci Bolognetti, Sapienza University, Rome 00161, Italy

## Abstract

Numb asymmetrically segregates at mitosis to control cell fate choices during development. Numb inheritance specifies progenitor over differentiated cell fates, and, paradoxically, also promotes neuronal differentiation, thus indicating that the role of Numb may change during development. Here we report that Numb nuclear localization is restricted to early thymocyte precursors, whereas timed appearance of pre-T-cell receptor (pre-TCR) and activation of protein kinase C*θ* promote phosphorylation-dependent Numb nuclear exclusion. Notably, nuclear localization of Numb in early thymocyte precursors favors p53 nuclear stabilization, whereas pre-TCR-dependent Numb nuclear exclusion promotes the p53 downmodulation essential for further differentiation. Accordingly, the persistence of Numb in the nucleus impairs the differentiation and promotes precursor cell death. This study reveals a novel regulatory mechanism for Numb function based on its nucleus–cytosol shuttling, coupling the different roles of Numb with different stages of T-cell development.

Cell fate decision of dividing progenitor-derived cells is a crucial event in development and diseases. Cell fate is often regulated by asymmetric cell division, which is a process by which progenitors asymmetrically segregate certain cell fate determinants during division, to generate two functionally different cells.^1,2^ The adaptor protein Numb was initially identified in *Drosophila* as a critical cell fate determinant,^3^ where loss of Numb and its homolog Numb-like results in the loss of neural progenitors, indicating that the presence of Numb is essential for maintaining the progenitors during the initial progenitor *versus* neural fate decision.^4,5^ However, re-expression of Numb is also required for further neural differentiation,^6,7^ indicating that the role of Numb in the same tissue may change over time.

Numb function in the immune system has been partially explored.^8,9^ Numb is involved in asymmetric division in hematopoietic stem cells,^10^ thymocytes^11^ and mature T lymphocytes.^12,13^ T cells develop from intrathymic CD4^−^CD8^−^ double-negative (DN) precursors that, after progression through DN1 (CD44^+^CD25^−^), DN2 (CD44^+^CD25^+^), DN3 (CD44^−^CD25^+^) and DN4 (CD44^−^CD25^−^), have to decide between proliferation, to increase the total number of precursors, or differentiation into CD4^+^CD8^+^ double-positive (DP) cells. This decision is made during DN3 stage and appears to be dependent on asymmetric segregation of Numb.^11^

As Numb is a well-characterized inhibitor of Notch-1 receptor signaling pathway,^14^ the ability of Numb to regulate cell fate decisions during development has been associated with this Numb function.^15^ However, the role of Numb during development could not be restricted to the control of Notch-1 signaling, as Numb has been implicated in the regulation of a variety of biochemical pathways, including the tumor suppressor p53.^16^ Increasing evidence suggests that p53 regulates cell differentiation in addition to cell proliferation, apoptosis and senescence.^17,18^

Notably, T-cell development is regulated by both Notch-1 and p53. Notch-1 signals appear to be critical for the very early steps of T-cell development (i.e. T-cell commitment).^19^ The involvement of p53 has been instead reported in the transition from the DN to the DP stage. However, while the overexpression of p53 during DN3 stage promotes a block in the differentiation and proliferation, resulting in a small thymus size,^20,21^ loss of p53 apparently does not affect thymocyte development, even though the vast majority of spontaneous malignancies in p53^−/−^ mice are lymphomas.^22^ Thus, the double function of Numb could be dependent on two different pathways, which may be differentially triggered during selected differentiation stages.

Recent data describe the presence of Numb in the nuclear compartment,^23^ besides its known cytoplasmic localization, raising the possibility that different Numb functions could be regulated by its differential subcellular localization. However, whether Numb may have different subcellular localizations in precursors or more differentiated T cell, how Numb import is regulated or how the nuclear localization affects its function during T-cell development remain unexplored. Here we show that Numb is an important regulator of p53 pathway during T-cell development, and we describe a novel molecular mechanism involved in the differential regulation of Numb–p53 axis based on the regulation of Numb nuclear import, emerging an interesting scenario where Numb can act as a regulator of two fundamental pathways during T-cell development.

## Results

### Pre-TCR signaling promotes Numb nuclear exclusion

**Figure 1:**
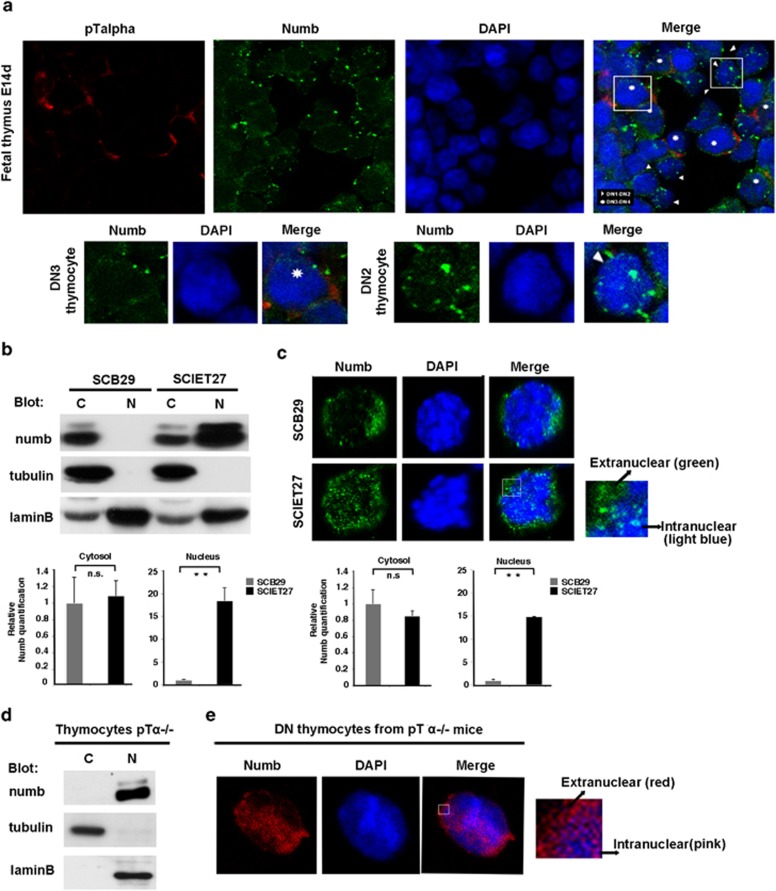
Numb subcellular distribution is dependent on pre-TCR. (**a**) Confocal images of wild-type (WT) fetal thymus E14d sections stained with antibodies against pT*α* (red) and Numb (green), and treated with 4',6-diamidino-2-phenylindole (DAPI), which stains the nucleus (blue). Asterisks indicate the pT*α*-positive cells and arrows indicate the pT*α*^−^ cells. (Lower panels) Higher magnification of a single DN3 thymocyte (left) and DN2 thymocyte (right). (**b** and **d**) Cytosolic and nuclear fractions in (**b**, upper panel) SCB29 and SCIET27 cells or (**d**) thymocytes derived from pT*α*^−/−^ mice were revealed in western blot with anti-numb antibody. Anti-tubulin and anti-laminB antibodies were used as the cytosol and nuclear markers, respectively. C, cytosolic fraction; N, nuclear fraction. (**c** and **e**) Confocal images of (**c**, upper panel) SCB29 and SCIET27 cells or (**e**) thymocytes derived from pT*α*^−/−^ mice stained with anti-numb (green or red), and treated with DAPI (blue). (**b** and **c**, lower panels) Relative Numb quantification of nuclear and cytosolic Numb: NS, nonsignificant; *P*-value >0.05. **P-value <0.02. All data are representative of three independent experiments

It has been recently shown that Numb localizes in the nucleus of breast cancer cell lines;^23^ however, no data about nuclear Numb localization in thymocytes have been reported. We focused our attention on the DN3 stage of thymocyte development, as we previously reported that DN3 development is dependent on Numb function.^11^ By confocal microscopy, we examined the subcellular localization of Numb using frozen sections of day 14 fetal thymi, where most of thymocytes are DN cells. We used an anti-pre-T-cell receptor-*α* chain (pT*α*) antibody to detect DN3-DN4 cells. As DN1 and DN2 thymocytes do not express pT*α*, negative cells were considered as DN1-DN2 thymocytes. Nuclear Numb was found only in pT*α*^−^ cells (DN1-DN2), where Numb was widely distributed in both the cytosol and the nucleus, whereas in pT*α-*positive cells (DN3-DN4), Numb was preferentially associated with the cell membrane and cytosol showing a nuclear exclusion (Figure 1a), suggesting that Numb nuclear localization is associated with early stages of T-cell development. Onset of pre-T-cell receptor (pre-TCR) signaling at DN3 stage is a crucial event during T-cell development, which regulates proliferation, differentiation and survival of progenitors. As Numb nuclear exclusion seems to be associated with DN3-DN4 stage, we suggest a key role for pre-TCR signaling in promoting nuclear exclusion of Numb. To test this hypothesis, we used the T-cell receptor-*β* (TCR*β*) chain-deficient cell line SCIET27, lacking pre-TCR, derived from severe-combined immunodeficient (SCID) mouse DN3 thymocytes,^24^ and the TCR*β* chain stably transfected daughter cell line SCB29, which express a functional pre-TCR. We analyzed the Numb localization by using nuclear and cytosolic fractionation assay. The efficiency of the subcellular fractionation method we used is shown in the Supplementary Figure 1. Interestingly, in the absence of pre-TCR, Numb was highly expressed in both nucleus and cytosol, whereas in the presence of pre-TCR, Numb appears to be preferentially localized in the cytosol, thus undergoing nuclear exclusion (Figure 1b). Consistently, these results were confirmed by the confocal microscopy analysis of Numb subcellular localization performed on the same cells (Figure 1c).

Moreover, in thymocytes derived from pT*α*^−/−^ mice, the absence of pre-TCR signaling results in an exclusive Numb nuclear localization (Figure 1d), although about 80% of the thymocytes were DN3 (CD25^+^CD44^−^). These data were also confirmed by confocal images, which show that CD44^−^ DN3-DN4 thymocytes from pT*α*^−/−^ mice display a high Numb nuclear localization (Figure 1e). Taken together, the data reported above demonstrate that Numb localizes in the nucleus of early thymocyte precursors and its nuclear localization changes over time, where the appearance of pre-TCR signaling at DN3 stage promotes nuclear exclusion.

### Pre-TCR signaling regulates PKC*θ*-mediated Numb phosphorylation

**Figure 2:**
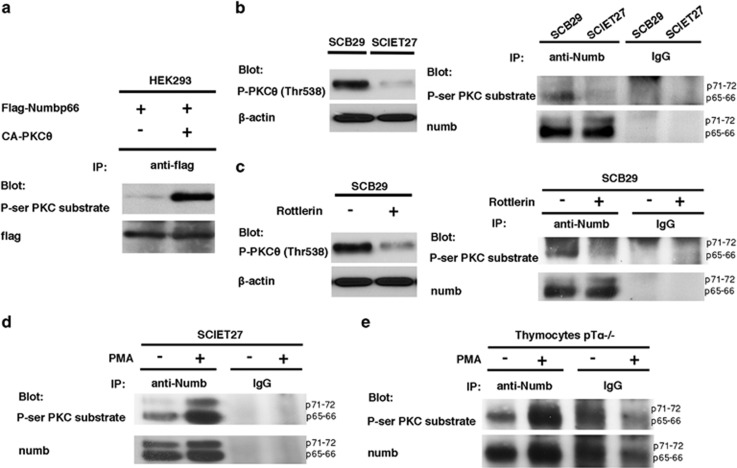
PKC*θ*-dependent Numb phosphorylation is regulated by pre-TCR. (**a**) Co-transfection of HEK293 cells with Flag-Numb p66 with or without CA-PKC*θ* expression plasmids. Whole-cell lysates were subjected to immunoprecipitation using anti-flag antibody and were revealed in western blot with anti-P-ser PKC substrate antibody. All samples were supplemented with empty vector so that the final concentration of DNA was the same in all reactions. (**b**, left panel) Total cell lysates of SCB29 and SCIET27 cells were resolved by western blot and incubated with anti-P-PKC*θ* (Thr538) and anti-*β*-actin antibodies. (Right panel) Endogenous Numb immunoprecipitates in whole-cell lysates of SCB29 and SCIET27 cells were incubated with anti-P-ser PKC substrate and anti-numb antibodies to confirm that equal Numb amounts were immunoprecipitated. (**c**, right panel) Numb immunoprecipitates in total lysates of non-treated or treated SCB29 cells with 3 *u*M of Rottlerin for 1 h were incubated with anti-P-ser PKC substrate and anti-numb antibodies. (Left panel) Total cell lysates from the same experiment were incubated with anti-P-PKC*θ* (Thr538) and anti-*β*-actin antibodies. (**d** and **e**) Numb immunoprecipitates in whole-cell lysates of (**d**) SCIET27 cells or (**e**) thymocytes from pT*α*^−/−^, treated or non-treated with 5 ng/ml of PMA for 1 h, were incubated with anti-P-ser PKC substrate and anti-numb antibodies. All samples were supplemented with empty vector so that the final concentration of DNA was the same in all reactions. All data are representative of three independent experiments

Phosphorylation status has been shown to regulate nuclear localization signal (NLS)-dependent nuclear import.^25^ Interestingly, atypical protein kinase C (aPKC)-mediated Numb phosphorylation has been described to regulate Numb subcellular localization and membrane polarization,^26^ influencing Numb function in neural^27,28^ and epithelial cells.^29^ The role of aPKC is not conserved in lymphocytes,^30^ whereas PKC*θ* has been shown to have key roles in T-cell activation and proliferation.^31^ Thus, we wanted to first analyze whether PKC*θ* is able to phosphorylate Numb. Human embryonic kidney 293 (HEK293) cells were co-transfected with Flag-Numb p66-expressing vector with or without a constitutively active mutant of PKC*θ* (CA-PKC*θ*). Immunoprecipitation of Flag-Numb p66 immunoblotted with a specific antibody, named anti-P-serine PKC (anti-*P*-ser PKC) substrate, which recognizes only the serine residues of substrate proteins phosphorylated by PKC kinases,^32^ shows that Numb p66 displays an increase in serine phosphorylation levels in the presence of CA-PKC*θ* (Figure 2a). As PKC*θ* activity is regulated by pre-TCR signaling,^33^ we next sought to determine whether Numb phosphorylation is influenced by the presence of pre-TCR. Using an antibody that recognizes the PKC*θ* phospho-threonine 538 (Thr538), which is phosphorylated only when PKC*θ* is activated,^34^ we found increased PKC*θ* activation in pre-TCR-competent SCB29 cells when compared with pre-TCR-deficient SCIET27 cells (Figure 2b, left panel). Accordingly, immunoprecipitation of endogenous Numb in SCB29 and SCIET27 cells shows an increment of phospho-serine-Numb in the presence of a functional pre-TCR (Figure 2b, right panel). To discard a redundant role between PKC isozymes expressed in thymocytes, we used Rottlerin, a compound that specifically inhibits PKC*θ* activity in T cells when it is used at a low concentration (3 *u*M).^35^ The phospho-Numb levels decreased in Rottlerin-treated SCB29 pre-TCR-competent cells when compared with cells treated with the vehicle alone (Figure 2c, left panel). The specificity of Rottlerin treatment was confirmed using the anti-P-PKC*θ* Thr538 antibody, which shows decreased PKC*θ* activity after treatment (Figure 2c, left panel). Conversely, in the absence of pre-TCR, the treatment with phorbol ester (phorbol-12-myristate-13-acetate (PMA)), a potent activator of PKC in eukaryotic cells, promotes an increase of phospho-Numb levels that correlates with an increase of the PKC*θ* activity^33^ (Figure 2d). Consistently, thymocytes from pT*α*^−/−^ mice showed low basal phosphorylation levels of Numb that were increased in the presence of PMA (Figure 2e). Taken together, our data demonstrate that Numb is a novel substrate of PKC*θ* in T-cell context and its phosphorylation is under the regulation of pre-TCR signaling.

### Phosphorylation regulates Numb nuclear import

**Figure 3:**
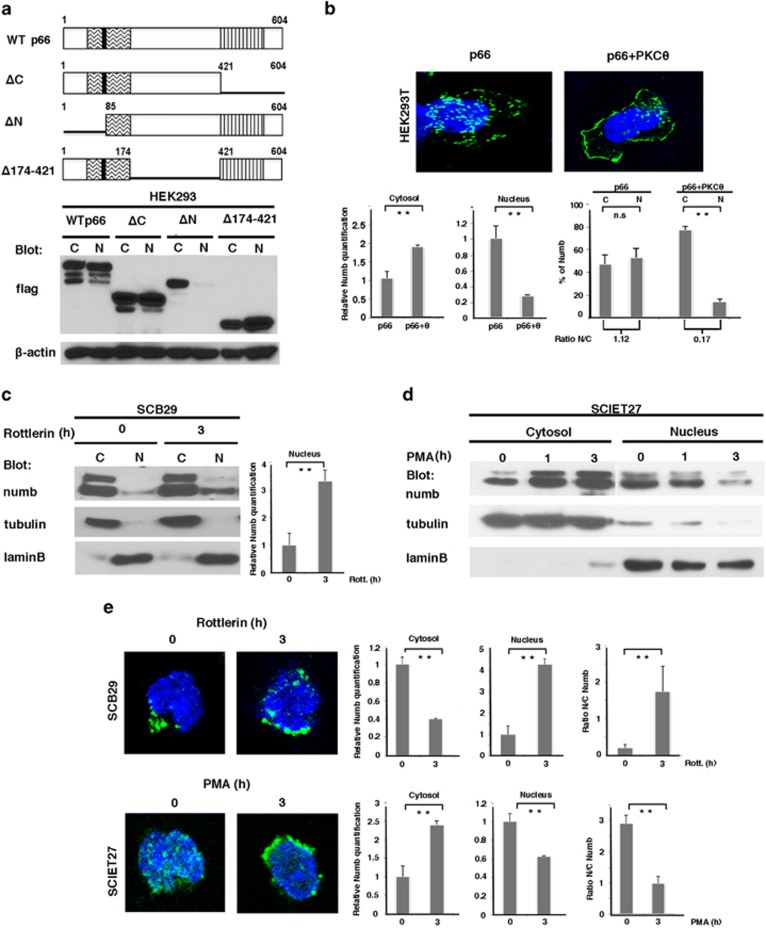
Regulation of Numb nuclear localization by PKC*θ*. (**a**, upper panel) Schematic representation of Numb mutants. All constructs are tagged with Flag. (Lower panel) Cytosolic and nuclear fractions of HEK293 cells transfected with Flag-Numb wild-type (WT) and Flag-Numb mutants were revealed in western blot with anti-flag and anti-*β*-actin antibodies. (**b**, upper panel) Confocal imagines of HEK293 cells transfected with Numb p66 with or without CA-PKC*θ* and stained with anti-flag (green) and 4',6-diamidino-2-phenylindole (DAPI) (blue) to detect nuclear Numb localization. (Lower panel) Relative Numb quantification of nuclear and cytosolic Numb or % of Numb in each compartment: NS, nonsignificant; *P*-value >0.05. **P-value <0.02. (**c** and **d**) Cytosolic and nuclear fractions of (**c**) SCB29 treated with Rottlerin or (**d**) SCIET27 treated with PMA at different times were revealed in western blot with anti-numb, anti-tubulin and anti-laminB antibodies. (**c**, right panel) Relative Numb quantification of nuclear Numb. **P-value <0.02. (**e**) Confocal images of SCB29 (upper panels) and SCIET27 (lower panels) cells stained with anti-numb (green) and DAPI (blue), treated or untreated with Rottlerin and PMA for 3 h, respectively. Right panels show the relative quantification of Numb in both the nucleus and cytosol, and the relative amount of Numb in the nucleus *versus* cytoplasm (ratio N/C). C, cytosolic fraction; N, nuclear fraction. Anti-tubulin and anti-laminB antibodies were used as markers of the cytosol and nucleus, respectively. All data are representative of three independent experiments

We then investigated the presence of NLS in Numb protein by using the program PSORT II. We identified two putative NLS sequences, both located in the N-terminal region of the protein including part of the phosphotyrosine-binding domain (PTB) domain (Supplementary Figure 2a). To determine the contribution of these putative NLS sequences to the nuclear import of Numb, we analyzed the ability of different Numb mutants to migrate into the nucleus (Figure 3a). Mutants containing the N-terminal region of the PTB domain were able to localize in the nucleus, whereas the truncation mutant lacking the N-terminal region (Numb-ΔN mutant), in which we deleted both the NLS sequences predicted by PSORT II, was defective in nuclear importing (Figure 3a, lower panel). Interestingly, we found a putative PKC-dependent phosphorylation site adjacent to the NLS at ninth position (Supplementary Figure 2b), raising the possibility that the subcellular localization of Numb is dependent on PKC*θ*-mediated phosphorylation. By confocal microscopy analysis, we analyzed the subcellular localization pattern of Numb transfected in HEK293 cells in the presence or absence of CA-PKC*θ*. As shown in Figure 3b, the co-transfection of CA-PKC*θ* results in a marked increase of cytosolic Numb and a decrease of nuclear Numb.

Confirming the above data, the inhibition of PKC*θ* by Rottlerin in a pre-TCR-competent context resulted in a change of Numb localization, promoting nuclear Numb accumulation (Figure 3c). Accordingly, an opposite effect was observed when PKC*θ* activity was mimicked in the absence of pre-TCR, by using PMA: while cytosolic Numb levels increase, nuclear Numb levels decrease markedly after PMA treatment of pre-TCR negative SCIET27 cells (Figure 3d). Consistently, the above results were also confirmed by confocal analysis (Figure 3e). Taken together, these data suggest that PKC*θ*-mediated Numb phosphorylation, once pre-TCR signaling is triggered at DN3 stage, results in the subcellular redistribution of Numb, promoting its nuclear exclusion.

### Numb nuclear localization is essential for p53 stabilization

**Figure 4:**
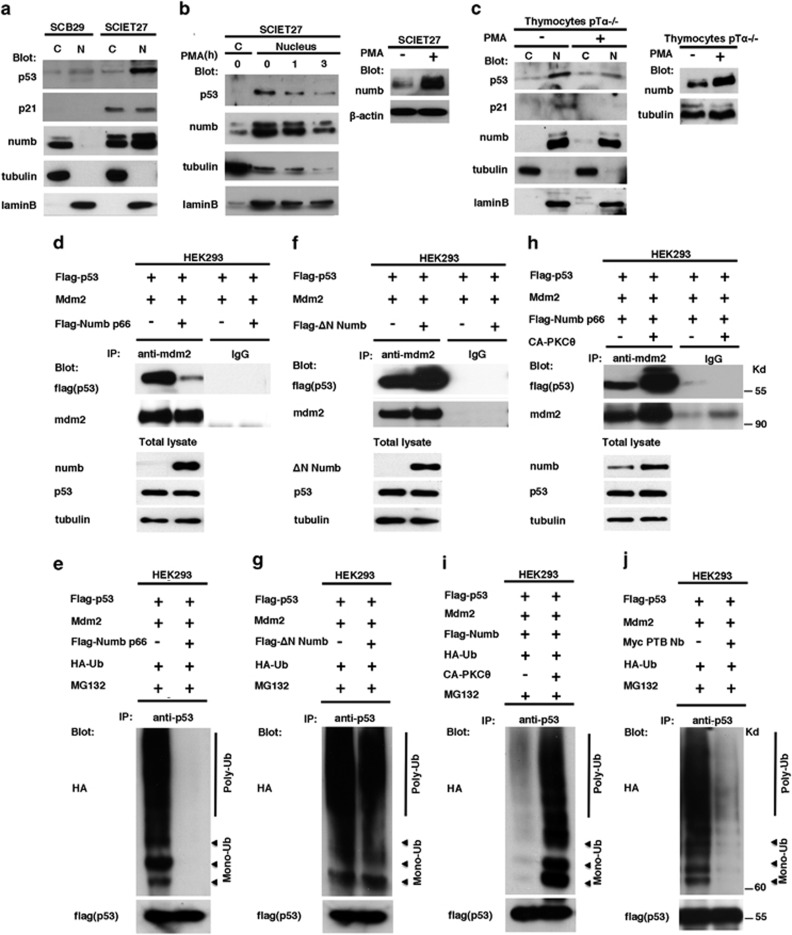
Numb nuclear localization regulates p53 stabilization. (**a**) Cytosolic and nuclear fractions of SCB29 and SCIET27 cells were revealed by western blot with anti-p53, anti-p21 and anti-numb antibodies. (**b** and **c**, left panels) Cytosolic and nuclear fractions of (**b**) SCIET27 cells and (**c**) pT*α*^−/−^ thymocytes treated with PMA at different times were revealed by western blot with anti-p53, anti-p21 and anti-numb antibodies. (Right panels) Total lysates of (**b**) SCIET27 cells and (**c**) thymocytes derived from pT*α*^−/−^ treated with PMA were revealed in western blot with anti-numb, anti-tubulin or anti-*β*-actin antibodies. (**d**, **f** and **h**) Whole-cell lysates of HEK293 cells transfected with Flag-p53 and Mdm2, (**d**) with or without Flag-Numb p66, (**f**) with or without Flag-Numb ΔN mutant or (**h**) with Flag-Numb p66 in the presence or absence of CA-PKC*θ*, were immunoprecipitated using an antibody against Mdm2 and were revealed in western blot with anti-flag (p53) and anti-Mdm2 antibodies. (Lower panels) Western blot of Numb and p53 on whole-cell extracts from (**d**, **f** and **h**). (**e**, **g**, **i** and **j**) Whole-cell lysates of HEK293 cells transfected with Flag-p53, Mdm2 and HA-ubiquitin, (**e**) with or without Flag-Numb p66, (**g**) with or without Flag-Numb ΔN mutant, (**i**) with Flag-Numb p66 in the presence or absence of CA-PKC*θ* or (**j**) with or without Myc-PTB Numb, and treated with 30 μM MG132 for 4 h, were immunoprecipitated using an anti-p53 antibody and revealed in western blot with anti-HA (against HA-flagged ubiquitin) and anti-p53 antibodies. All samples were supplemented with empty vector so that the final concentration of DNA was the same in all reactions. C, cytosolic fraction; N, nuclear fraction. Anti-tubulin and anti-laminB antibodies were used as markers of the cytosol and nucleus, respectively. All data are representative of three independent experiments

It has been shown that Numb promotes p53 stabilization blocking the function of the mouse double minute 2 (Mdm2) E3-ubiquitin ligase and, interestingly, both p53 and Mdm2 are hosted in the nucleus.^36^ Western blot analysis in SCB29 and SCIET27 cells revealed that Numb nuclear levels were correlated with p53 levels, resulting in increased nuclear p53 and its target p21 in SCIET27 cells, in which we observe nuclear Numb, when compared with SCB29 cells, displaying Numb nuclear exclusion (Figure 4a). Moreover, treatment of SCIET27 cells with PMA, which activates PKC*θ* and decreases Numb nuclear levels, was also able to promote a decline in p53 level in the nucleus (Figure 4b). Notably, Numb total levels increased after PMA treatment, suggesting that p53 stabilization was dependent on nuclear Numb levels but not on total Numb levels (Figure 4b, right panel). Accordingly, similar results were obtained in thymocytes derived from pT*α*^−/−^ mice (Figure 4c). Next, we analyzed p53 stability under different Numb localization conditions. Figure 4d shows that overexpression of Numb in HEK293 cells disrupts p53–Mdm2 interaction, resulting in a decrease of p53 poly- and monoubiquitination after treatment with the proteasome inhibitor MG132 (Figure 4e). In contrast, we observed that upon Numb nuclear exclusion conditions, p53 does not undergo stabilization. First, when ΔN-Numb mutant, which shows nuclear exclusion (Figure 3a), was expressed, Mdm2–p53 interaction and p53 ubiquitination were comparable with that observed in the absence of ΔN-Numb (Figures 4f and g), although ΔN mutant maintained the ability to bind to Mdm2 and to be degraded as the WT Numb (Supplementary Figures 3a and b). Second, coexpression of Numb and PKC*θ*-CA, which results in Numb nuclear exclusion, was not able to disrupt Mdm2–p53 complex or to decrease p53 ubiquitination after MG132 treatment (Figures 4h and i). Next, we tested whether the modifications of p53 ubiquitination observed in the above experiments were linked with its stabilization using the protein synthesis inhibitor cycloheximide (CHX), to avoid the effect of new synthetized factors. In these experimental conditions, neither the overexpression of WT Numb together with CA-PKC*θ* nor the one that of ΔN-Numb mutant alone (Supplementary Figure 3c, lanes 3 and 4) was able to stabilize p53 protein, resulting in the same degradation rate than in the absence of Numb (Supplementary Figure 3c, lane 1), whereas in the presence of overexpressed WT Numb alone (Supplementary Figure 3c, lane 2), p53 was completely stabilized, as expected. Taken together, these results show that Numb nuclear import is essential for Numb-mediated p53 stabilization.

Notably, Numb PTB peptides have been used to block Mdm2 function and consequently to stabilize p53-mimicking Numb function.^37^ We confirm here that Numb PTB mutant retains the ability to interact with Mdm2 (Supplementary Figure 4a). In addition, overexpression of Numb PTB was able to stabilize p53, by decreasing its ubiquitination in comparable manner, with respect to WT Numb overexpression (Figure 4j). Interestingly, Numb PTB nuclear localization was independent of PKC*θ* activity, as CA-PKC*θ* did not affect the subcellular localization pattern of PTB mutant (Supplementary Figure 4b). Taken together, these data show that Numb nuclear function is dependent on the PTB domain. Moreover, Numb PTB nuclear localization appears not to be regulated by PKC*θ*, possibly because of its small size (18 kDa), which may allow the cross of nuclear membrane by diffusion.^38^

### Nuclear localization of Numb promotes its own proteosomal degradation

As the turnover of Numb, similar to p53, is also regulated by Mdm2,^37^ one likely cause of increase in Numb total levels in the presence of activated PKC after PMA treatment, which increases cytosolic levels of Numb (Figures 4b and c), is that the half-life of Numb could be coupled with its nuclear localization, being dependent on its interaction with Mdm2.

**Figure 5:**
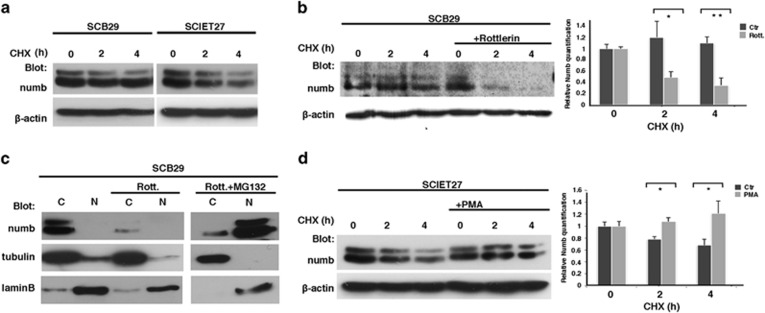
Nuclear degradation of Numb. (**a**) SCB29 or SCIET27 cells were treated with CHX in a time-course study (0–2–4 h) before lysis. Whole-cell lysates were revealed in western blot with anti-numb and anti-*β*-actin antibodies. (**b**, left panel) SCB29 cells were treated with CHX at different times in the absence or presence of Rottlerin and whole cell lysates were revealed in western blot with anti-numb and anti-*β*-actin antibodies. (Right panel) Relative quantification of Numb modulation determined by optical densitometry (OD); quantification was normalized using *β*-actin levels. (**c**) Nuclear and cytosolic fractions of SCB29 cells treated with Rottlerin (Rott.) or DMSO, or Rottlerin plus MG132 for 12 h were revealed in western blot with anti-numb antibody, using anti-tubulin and anti-laminB antibodies as the cytosol and nucleus markers, respectively. (**d**, left panel) SCIET27 cells were treated with CHX at different times in the absence or presence of PMA and whole cell lysates were revealed in western blot with anti-numb and anti-*β*-actin antibodies. (Right panel) Relative quantification of Numb modulation determined by OD. **P-value <0.02 and *P-value <0.05. C, cytosolic fraction; N, nuclear fraction. All data are representative of three independent experiments

However, nuclear Numb degradation has not been reported before. To address this issue, we compared Numb half-life in SCB29 and SCIET27 cells, in which Numb display a differential subcellular localization pattern. Our analysis revealed a correspondence between the localization and the degradation rate of Numb. Indeed, SCB29 cells, where Numb was cytosolic, displayed a higher Numb stability, whereas in SCIET27 cells, where Numb was localized in the nucleus, Numb was rapidly degraded (Figure 5a). Pharmacologic treatments, which decrease or increase PKC*θ* activity, changed Numb localization pattern and impact in the half-life of Numb; when SCB29 cells were treated with Rottlerin in the presence of CHX, there was a significant decline in the half-life of Numb protein (Figure 5b). Interestingly, longer exposition to Rottlerin markedly decreased both cytosolic and nuclear levels of Numb in SCB29 cells and this effect was reversed in the presence of MG132, which revealed a Numb accumulation in the nuclear compartment (Figure 5c), showing that the downmodulation of Numb is because of its nuclear proteosomal degradation. The opposite effect was observed in SCIET27 cells treated with PMA, where the half-life of Numb displayed an increase (Figure 5d).

### Enforced Numb nuclear function during DN3 stage inhibits T-cell precursors' differentiation

**Figure 6:**
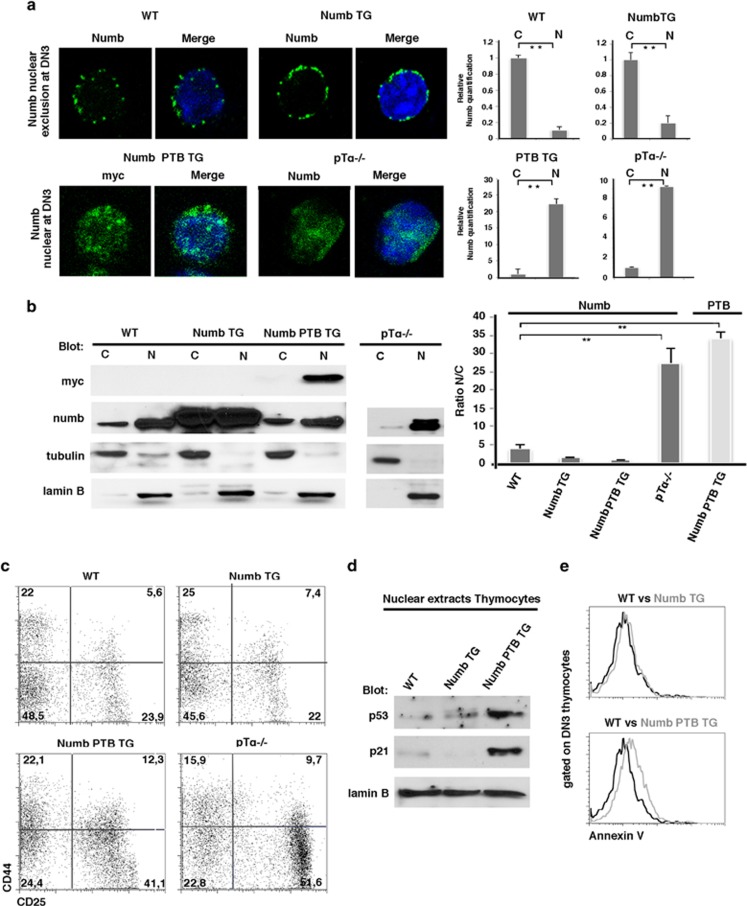
Numb localization regulates DN3 differentiation and death. (**a**, left panel) Confocal imagines of DN thymocytes sorted from wild-type (WT), Numb TG, Numb PTB TG and pT*α*^−/−^ mice and stained with anti-CD44 (red), anti-numb (green), anti-myc (green) (to reveal myc-tagged Numb PTB) and 4',6-diamidino-2-phenylindole (DAPI) (blue). (Right panel) Relative quantification of cytosolic or nuclear Numb. (**b**, left panel) Cytosolic and nuclear extracts from WT, Numb TG, Numb PTB TG and pT*α*^−/−^ thymocytes were resolved by western blot with anti-numb and anti-myc antibodies. Anti-tubulin and anti-laminB antibodies were used as the cytosol and nucleus markers, respectively. (Right panel) Relative quantification of Numb (black bars) or Numb PTB (gray bars) nuclear/cytosolic ratio determined by optical densitometry (OD); quantification was normalized using tubulin and laminB levels. **P-value <0.02. (**c**) CD25^+^ and/or CD44^+^ subset distribution of DN thymocytes from WT, Numb TG, Numb PTB TG and pT*α*^−/−^ mice. Quadrants represent DN1, DN2, DN3 and DN4 sub-populations (clockwise from top left to bottom left) and the numbers represent the percentage of each sub-population. (**d**) Nuclear extracts from WT, Numb TG and Numb PTB TG mice were resolved by western blot with anti-p53, anti-p21 and anti-laminB antibodies. (**e**) Overlay of Annexin V flow cytometric analysis of DN3 thymocytes from Numb TG and Numb PTB TG mice, compared with DN3 thymocytes from WT mice. The Annexin V MFI were calculated for DN3 cells from WT=20.4, Numb TG=24.9 and Numb PTB TG=43.1. All data are representative of three independent experiments

Next, we analyzed the localization of Numb during DN3 stage, after a fluorescent-activated cell sorting of total DN thymocytes derived from WT Numb, transgenic mice overexpressing full-length Numb (Numb TG) and transgenic mice overexpressing the myc-tagged Numb PTB domain (Numb PTB TG).^11^ As DN1-DN2 thymocytes are CD44 positives and DN3-DN4 are CD44 negatives, we performed a CD44 staining of isolated DN cells to discriminate between these sub-populations. Confocal images revealed that overexpression of full-length Numb in Numb TG did not affect the subcellular localization of Numb at DN3 stage (CD44^−^ cells) when compared with WT thymocytes, maintaining the nuclear exclusion at this stage. Conversely, Numb PTB retained the ability to localize in the nucleus during DN3 stage despite the presence of pre-TCR, confirming our results above (Figure 6a), and it was comparable to that one of full-length Numb in thymocytes from pT*α*^−/−^ mice, being mostly nuclear (Figure 6a). These data were also confirmed by using nuclear–cytosol fractionated extracts from total thymocytes derived from the same mice (Figure 6b). We further analyzed the thymus phenotype of different mutant mice. Interestingly, phenotype analysis of thymocytes from Numb PTB transgenic and pT*α*^−/−^ mice revealed a correlation between the presence of Numb or Numb PTB in the nucleus at DN3 stage and the increased percentage in CD25^+^ CD44^−^ DN3 cells and decreased percentage of CD25^−^ CD44^−^ DN4 cells, suggesting a block in T-cell differentiation at DN3 stage. Conversely, nuclear exclusion of Numb at DN3 stage in WT and Numb TG mice correlated with the predicted normal T-cell development and differentiation observed in these mice (Figure 6c). In keeping with this, we observed that while overexpression of WT Numb in Numb TG thymocytes does not alter the expression levels of p53 and p21, when compared with wild-type thymocytes, overexpression of Numb PTB instead increased both p53 and p21 nuclear levels in thymocytes (Figure 6d), similar to what we observed in pre-TCR loss conditions. Moreover, the increased p53 and p21 levels observed correlate with an increased cell death by apoptosis of DN3 cells measured by Annexin V staining (Figure 6e). Therefore, inhibition of Numb nuclear function mediated by pre-TCR signaling appears to be essential during DN3 stage to promote proper thymocytes survival and differentiation.

## Discussion

**Figure 7:**
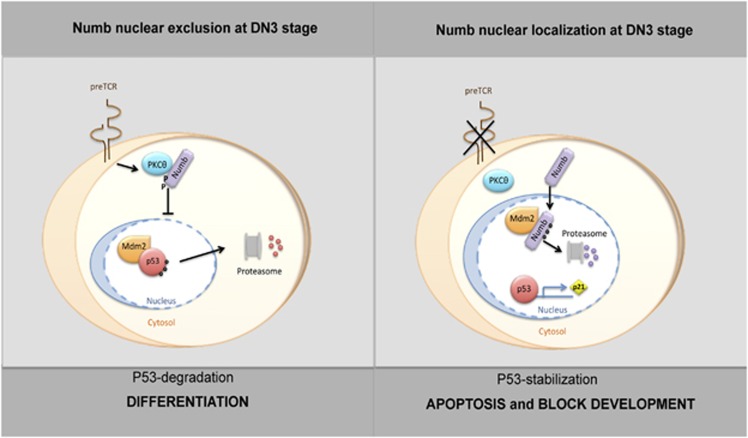
Numb and p53 relationship in T-cell development. The schematic diagram shows how the different subcellular localization patterns of Numb, depending on the presence of pre-TCR, may reciprocally regulate p53 and its own stability. Left panel illustrates DN3 thymocytes, in which the presence of the pre-TCR signal promotes PKC*θ*-mediated Numb phosphorylation and nuclear exclusion. This event results in the nuclear Mdm2-dependent p53 ubiquitination and degradation, finally allowing the DN3 cell survival and differentiation. Right panel shows mutant DN3 thymocytes in which the absence of pre-TCR signal does not allow PKC*θ*-dependent phosphorylation of Numb, which localizes mainly in the nucleus interfering with the Mdm2–p53 complex formation. This leads to proteasome-dependent degradation of Numb itself and p53 stabilization, finally resulting in both cell death and developmental block of thymocytes

During development, Numb exerts two different roles that may seem opposite, as it maintains progenitor fate and promotes cell differentiation.^6^ This makes it difficult to understand which pathways are regulated by Numb and raises the possibility that these different roles are mediated by diverse Numb functions. In this report, we demonstrate that Numb extends its capacity to the regulation of p53 function during T-cell development. Our data provide evidence for a model, where the different subcellular localization patterns of Numb may reciprocally regulate p53 and its own stability. In this model, the presence and function of pre-TCR signaling at DN3 stage, by promoting the nuclear exclusion of Numb, is responsible of the p53 downmodulation, necessary to allow the survival of DN3 thymocytes, and thus to continue their differentiation (Figure 7). This is in agreement with previous reports where p53 downmodulation has been suggested to be a pre-TCR triggering downstream event.^20,21^ Notably, loss of pre-TCR signaling in SCID, RAG^−/−^ and CD3*γ*^−/−^ mice^21^ promotes a block of thymocyte differentiation at DN3 stage, associated with an increase of p53 levels. In keeping with this, our data show that the absence of pre-TCR signaling in thymocytes from pT*α*^−/−^ mice promotes a nuclear Numb accumulation at DN3 stage, which result in high levels of p53, a block at DN3 stage, an increase in cell death by apoptosis, and consequently a small thymus size. Interestingly, Numb PTB TG mice, where nuclear Numb function is maintained during DN3 stage, show a similar phenotype. Notably, Mdm^2 puro/Δ7−12^ mice,^39^ where the level of Mdm2 expression is decreased, show an increment of p53 levels associated with a block of thymocyte differentiation similar to that one observed in Numb PTB TG mice. Moreover, high levels of p53 expression are observed in thymocytes of embryonic thymus from E14.5 to E16.5.^40^ Notably, we show that the high levels of p53 expression in the thymus of E14.5 mice correlate with the presence of nuclear Numb in such age thymus. All these data suggest that Numb nuclear function during T-cell development is finely regulated by its localization. However, it appears to be independent of total Numb levels as we observed that overexpression of Numb in Numb TG mice does not affect its nuclear function. This is also in agreement with previous data where overexpression of Numb does not influence cell survival.^41^ Our findings suggest that the main role of Numb in T-cell development could be focused on p53 regulation. In this scenario, the absence of Numb is expected to impair p53 stabilization but not to alter T-cell specification, as previous data have shown,^9^ as loss of p53 apparently does not affect thymocyte development.^22^

Data from other cell systems in which Numb function has been studied in detail reveal that the dual role of Numb is regulated based on its interaction with ACBD3, a Golgi-associated protein.^7^ ACBD3 has been described to hold its targets within cytosol during mitosis, by inhibiting their translocation into the nucleus.^42^ Moreover, Numb AB domain is responsible of Numb–ACBD3 interaction. Notably, we show here that the NLS of Numb is hosted in this domain, thus raising the interesting possibility that cytosolic ACBD3 may impinge on Numb nuclear translocation, finally inhibiting p53 activity and consequently promoting a change in Numb function over time. Taken together, these observations allow us to hypothesize that a similar mechanism may be active during thymocyte differentiation, although the ACBD3 expression has not been reported in lymphocytes, as yet. Notably, Igl mutant brains show an increase in cortical aPKC activity, resulting in the deregulation of Numb phosphorylation. Under these conditions, neuroblasts are not able to complete the differentiation process.^27^ Interestingly, the phenotype exhibited by Igl mutant is remarkably similar to that observed in ACBD3 mutants, in which inhibition of nuclear function of Numb may be responsible of an altered differentiation.

Overall, our data reveal important keys in the Numb function regulation, where Numb function is exquisitely regulated through its own subcellular distribution to balance alternative pathways at different stages of development.

## Materials and Methods

### Mice

pT*α* ^−/−^,^43^ Numb TG and Numb PTB TG mice^11^ have been described elsewhere. The studies involving animals were conducted following Italian National guidelines for animal care established in the Decree number 116 of 27 January 1992, in accord with the Directive CEE 86/609, as well as in the Circular number 8 of the Ministry of Health of 23 April 1994.

### Flow cytometry

Thymocytes were washed two times in PBS and stained at 4 °C with antibodies, anti-CD44, anti-CD4, anti-CD8 and anti-CD25 (BD Bioscience, San Jose, CA, USA). For Annexin V (BD Bioscience) analysis, the manufacturer's instructions were used. Analysis was performed on a FACSCalibur (BD). Files were analyzed with FlowJo Version 4.6.2 software (TreeStar Inc., Ashland, OR, USA).

### Immunofluorescence and confocal microscopy

Fetal thymi were fixed with 4% paraformaldehyde solution in PBS, treated with 20% sucrose solution in PBS, immersed in OCT (Tissue Tek) and frozen with a mixture of 2-methylbutene and dry ice. Sections (10 *μ*m) were fixed, permeabilized with Triton X-100, blocked in a solution containing 5% normal goat serum and 3% of bovine serum albumin and stained in the same solution. Sections were mounted with ProLong Antifade (Molecular Probes, Life Technologies Corporation, Carlsbad, CA, USA). Antibodies used were as follows: anti-numb (Cell Signaling, Beverly, MA, USA), anti-CD44 Alexa-647 conjugated, anti-pT*α* (BD Bioscience), biotinylated anti-myc (Upstate Biotechnology) and DAPI. We use a Leica TCS SP5 confocal microscope with either x20 or x40 objective; images were collected at 8-bit depth, with a resolution of 1024 × 1024 pixels. Images were processed using LAS AF (Leica Microsystems) and Adobe Photoshop software (Adobe Systems, San Jose, CA, USA). The software LAS AF (Leica Microsystem) was used to quantify fluorescent images. Inside each cell, one gate for the nucleus, using DAPI as a marker, and one gate for the cytosol was drawn and threshold intensities of total pixels were determined for Numb.

### Plasmids and cell transfection

Transient transfection experiments were performed by TransFectin Lipid Reagent (Bio-Rad, Hercules, CA, USA) according to the manufacturer's instructions. The expression vectors for CA-PKC*θ*,^44^ Flag-WT Numb and Flag-Numb mutant,^45^ Myc-PTB Numb (provided by M Canelles), Flag-p53, Mdm2 and HA-ubiquitin have been described elsewhere.

### Cells lines and treatments

HEK293 cells were used for transient transfection experiments. SCIET27 (TCR*β*-deficient) and SCB29 (TCR*β*-transfected) cells^24^ were described previously. In similar cases, cells were treated with different compounds. Thirty micromolar proteasome inhibitor MG132, 3 *μ*M PKC*θ* inhibitor Rottlerin (Calbiochem, Darmstadt, Germany), 10 *μ*g/ml ribosome inhibitor CHX (Sigma-Aldrich, St. Louis, MO, USA), 7.5 ng/ml nuclear export inhibitor leptomycin B (Santa Cruz Biotechnology, Santa Cruz, CA, USA) and 50 ng/ml PKC activator PMA (Sigma-Aldrich) for the times indicated.

### Protein extracts, immunoprecipitation and immunoblotting

Total extracts and immunoprecipitations were performed as described previously.^33^ For immunoblotting, proteins were resolved by SDS-PAGE and blotted into the nitrocellulose membrane. The blots were incubated with the following antibodies: anti-*β*-actin, anti-HA, anti-myc and anti-flag were from Sigma-Aldrich; anti-tubulin, anti-p53, anti-p21, anti-Mdm2 and anti-laminB were from Santa Cruz Biotechnology; and anti-numb, anti-phospho-PKC*θ* (Thr538) and anti-P-ser PKC substrate were from Cell Signaling.

### Subcellular fractionation

Cells were resuspended in ice-cold hypotonic buffer (10 mM KCl, 10 mM HEPES (pH 7.4), 10 mM NaCl, 0.1 mM EDTA, 1 mM DTT and 0.5 mM PMSF) and incubated on ice for 15 min. A measure of 0.6% NP-40 was added and lysates were vortexed for 10 s. The lysates were centrifuged at 12 000 r.p.m. for 1 min to remove nuclei and cell debris, and the supernatant (cytosol) was collected and centrifuged at 12 000 r.p.m. for 30 min at 4 °C. The pellet was resuspended in hypotonic buffer with 0.6% of NP-40 and vortexed and washed three times at 12 000 r.p.m. for 2 min at 4 °C. The pellet was resuspended in the buffer (0.4 M NaCl, 20 mM HEPES (pH 7.4), 1 mM EDTA, 1 mM DTT and 1 mM PMSF) and incubated on ice for 30 min. The lysates were centrifuged at 12 000 r.p.m. for 30 min and the supernatant (nucleus) was collected.

## Abbreviations

pre-TCR: pre-T-cell receptor
PKC*θ*: protein kinase C theta
DN: double-negative cells CD4^−^/CD8^−^
DP: double-positive cells CD4^+^/CD8^+^
Pt*α*: Pre-T-cell receptor alpha chain
TCR*β* chain: T-cell receptor-*β* chain
SCID: severe-combined immunodeficiency
a-PKC: atypical protein kinase C
CA-PKC*θ*: active mutant PKC*θ*
PMA: phorbol-12-myristate-13-acetate
NLS: nuclear localization signal
PTB: phosphotyrosine-binding domain
Mdm2: mouse double minute 2
CHX: cycloheximide
