# Supplementary material for: Numb-dependent integration of pre-TCR and p53 function in T-cell precursor development
Source: Cell Death Dis. 2014 Oct 16;5(10):e1472–. doi: 10.1038/cddis.2014.438 (PMC4237259; doi:10.1038/cddis.2014.438)
Supplement: Supplementary Figures [file cddis2014438x1.pdf]

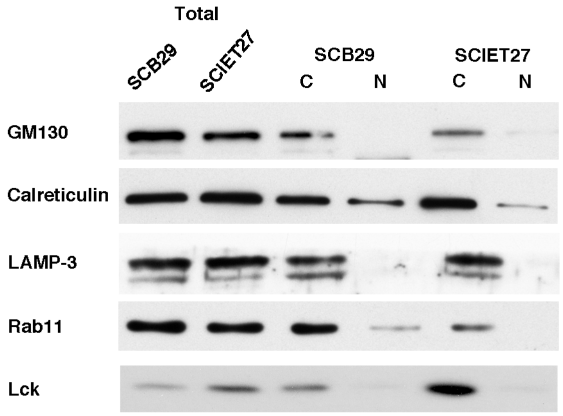


**Figure Supplementary 1.** Total lysate and fractionated extracted from SCB29 and SCIET27 were revealed by western blot with anti-Rab11 GTPase (early-recycling endosome marker), LAMP3 (lysosome marker), anti-Calreticulin (reticulum endoplasmatic marker), anti-Lck (cytoplasmatic membrane marker) and anti- GM130 (Golgi marker) antibodies.

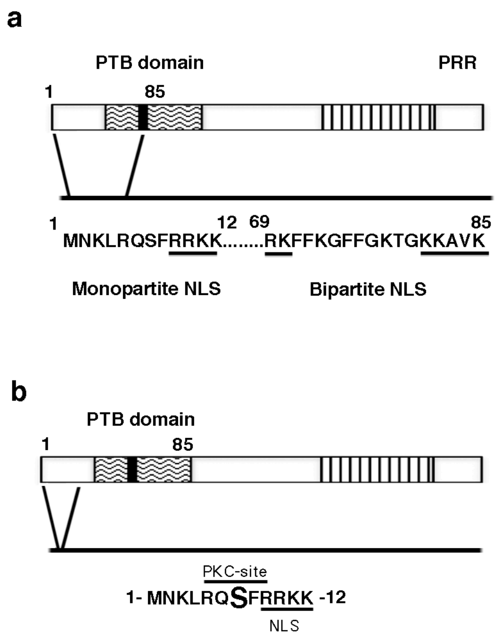


**Figure Supplementary 2.** (a) PSORTII analysis of Numb predicts two nuclear localization signals in the N-terminal region, one monopartite $^{9}$RRKK$^{12}$ and one bipartite $^{69}$RK$^{70}$---(10aa)----$^{81}$KKAVK$^{85}$. (b) Schematic representation of WT Numb showing the putative PKC-phosphorylation site (serine residues surrounded by Arg or Lys at the -2 and +2 positions and a hydrophobic residue at the +1 position)

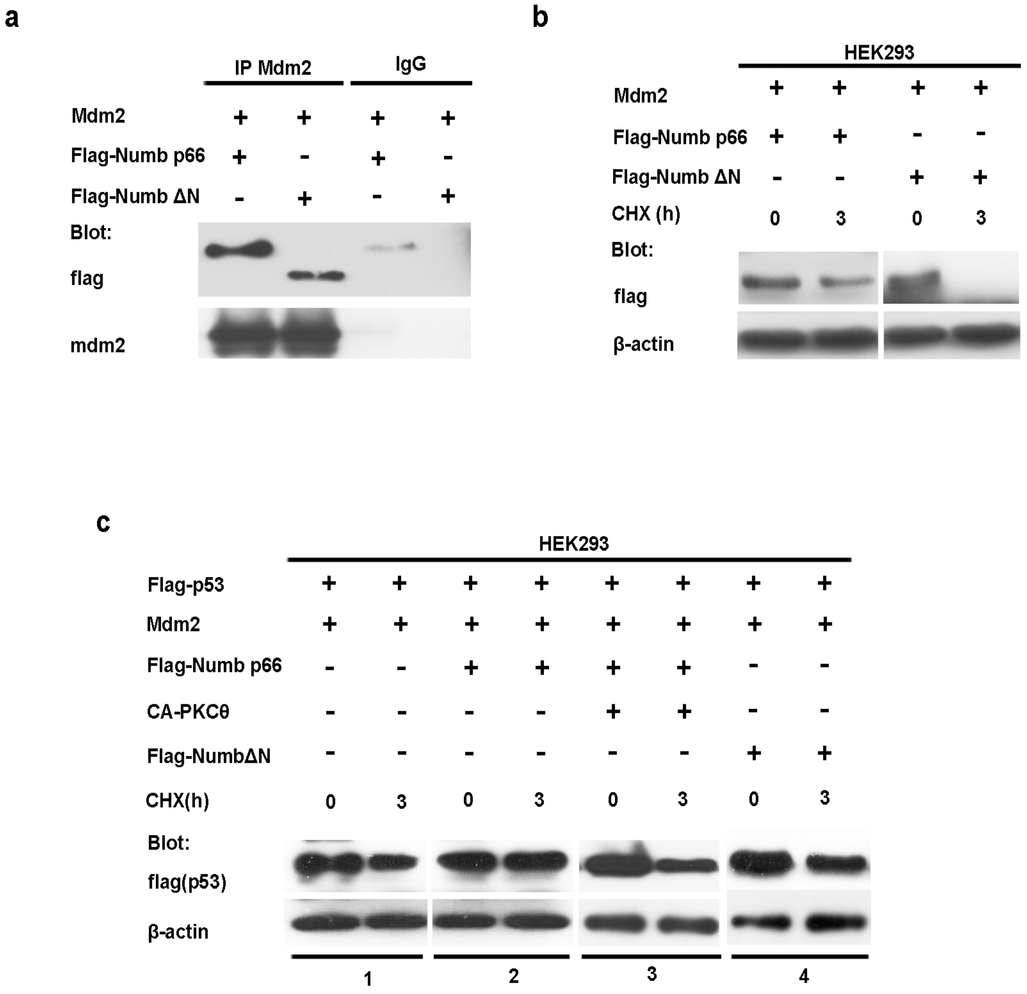


**Figure Supplementary 3.** Numb ΔN mutant interacts with Mdm2 without stabilizing p53. (a) HEK293 cells, co-transfected with Mdm2 and Flag-Numb p66 or Flag Numb ΔN mutant. Mdm2 immunoprecipitation was revealed in western blot with anti-flag antibody and anti-Mdm2 antibodies as immunoprecipitation control. (b) Co-transfected HEK293 cells with Mdm2 and Flag-Numb p66 or Flag-Numb ΔN expression plasmids, were treated with cicloheximide (CHX) for different times before lysis. Whole cell lysates were revealed in western blot with anti-flag and anti-β-actin antibodies. (c) HEK293 cells, co-transfected with Mdm2 and Flag-p53, with or without Flag-Numb p66, CA-PKCθ and Flag-Numb ΔN mutant, were treated with cicloheximide for different times before lysis. Whole cell lysates were revealed in western blot with anti-flag and anti-β-actin antibodies. All data are representative of three independent experiments.

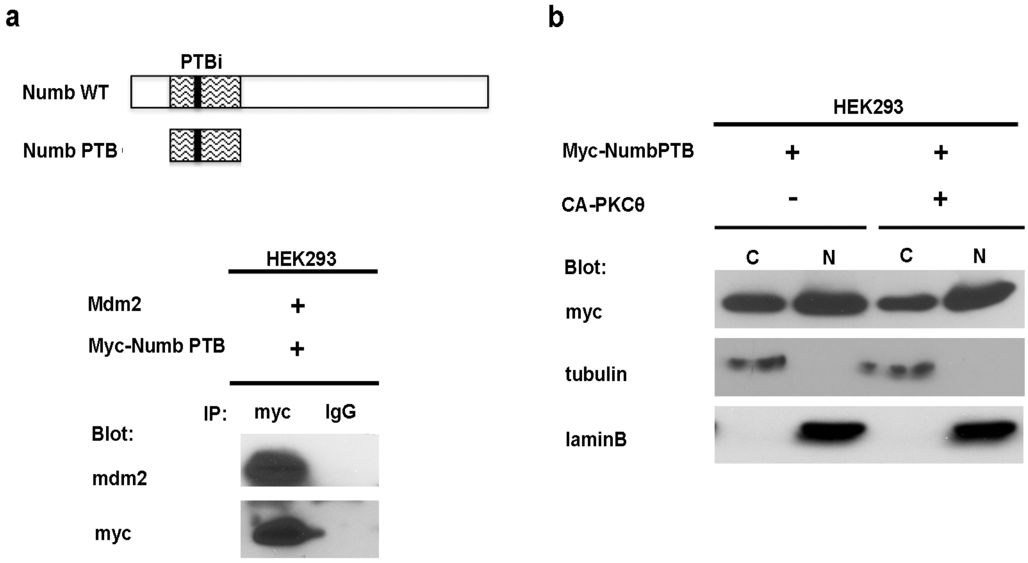


**Figure Supplementary 4.** Numb PTB mutant interact with Mdm2 and localizes constitutively in the nucleus. (a) Top panel, schematic representation of PTB Numb mutant. Bottom panel, myc-PTB immunoprecipitation in HEK293 cells co-transfected with Mdm2 and myc-PTB was revealed in western blot with anti-Mdm2 and anti-myc (to reveal myc-tagged PTB) antibodies. (b) Cytosolic and nuclear fractions of HEK293 cells co-tranfected with myc-PTB, with or without CA-PKCθ, were revealed in western blot with anti-myc, anti-tubulin and anti-laminB antibodies. All samples were supplemented with empty vector so that the final concentration of DNA was the same in all reactions. C, cytosolic fraction. N, nuclear fraction. All data are representative of three independent experiments.
